# Non-Invasive Characterization of the Pancreas During Bariatric Surgery *via* Circulating Pancreatic Specific Cell-free Messenger RNA

**DOI:** 10.3389/fgene.2021.742496

**Published:** 2021-10-11

**Authors:** Kong Kiat Whye, E ShyongTai, Asim Shabbir, Chin Meng Khoo, Winston Koh

**Affiliations:** ^1^ Molecular Engineering Lab, Institute of Molecular and Cell Biology, A*STAR, Singapore, Singapore; ^2^ Division of Endocrinology, Department of Medicine, National University Hospital, Singapore, Singapore; ^3^ Yong Loo Lin School of Medicine, National University of Singapore, Singapore, Singapore; ^4^ Department of Surgery, National University Hospital, Singapore, Singapore

**Keywords:** cfmRNA, liquid biospsy, bariatric surgery, pancreas, metabolic health

## Abstract

Bariatric surgery results in sustained weight loss and improvement in glucose homeostasis. However, the lack of accessible non-invasive tools to examine molecular alterations occurring in the pancreas limits our understanding of the causes and recovery of glucose homeostasis. Here, we describe the use of a circulating cell free mRNA (cfmRNA) based multiplex qPCR assay to selectively amplify and quantify circulating pancreatic specific transcripts levels within plasma. We applied this assay to a cohort of 58 plasma samples consisting of 10 patients that tracks multiple time points including pre and post-bariatric surgery. In our targeted multiplex screen of 14 selected pancreatic specific circulating transcripts, we identified 13 pancreatic specific transcripts that can be amplified from plasma. Furthermore, when quantifying the amplicons obtained in the short-term post-surgery (2 weeks–1 month) and long-term (3–12 months), we observed a consistent reduction of circulating GCG transcripts during short term post-surgery. Across the cohort, GCG cfmRNA levels correlated significantly with common metrics of improvement following bariatric surgery such as: haemoglobin A1c levels (R: −0.41, *p*-value: 0.0039) and percentage of excess weight loss (R: 0.29, *p*-value: 0.046).

## Introduction

After bariatric surgery, patients exhibit drastic improvements in metabolic health as reflected in their loss of body weight/BMI, and reversal from diabetes mellitus. The reversal in diabetes mellitus suggests an improvement to pancreatic beta-cell function/health (Bradley et al., 2012). However, changes in molecular function of pancreatic islets that resulted in these improvements remains an area of great uncertainty. Recent single cell RNA sequencing studies using donor pancreases obtained from deceased individuals of different BMI and obesity levels, have revealed transcriptional changes within α- and β-cell types that directly correlate to BMI (Segerstolpe et al., 2016). When taken in consideration the significant reduction in BMI following bariatric surgery, reversal in gene expression can be expected from these pancreatic cell types. Unfortunately, it is almost impossible to access pancreatic tissues/cells from living patients to study the cellular changes with weight loss, even less so when considering the study to examine the trajectories of cellular changes across multiple time points following surgery.

Liquid biopsies, that use circulating mRNA in plasma, have recently been demonstrated as an alternative way of non-invasive examination of molecular alterations in hard-to-reach tissues like the brain (Koh et al., 2014; Toden et al., 2020). We hypothesize that liquid biopsies that specifically targets circulating pancreatic mRNA transcripts improvements could be employed to capture changes in pancreatic health in patients undergoing bariatric surgery.

In our study, we begin with human protein atlas (Pontén et al., 2008; Uhlén et al., 2015) that maintains tissue-enriched gene lists which is defined as having at least four-fold higher average mRNA level in a group of 2–5 tissues compared to any other tissues in the atlas. Starting with the pancreatic enriched gene list, we were able to design multiplex primers for 14 targets: KLK1, CTRB1, ERP27, IAPP, PRSS1, CELA3A, PLA2G1B, PNLIP, CUZD1, CPB1, CPA1, CLPS, INS, GCG which we subsequently amplified for quantification with real-time qPCR. This assay is used to track temporal changes in a cohort of 58 plasma samples drawn from 10 patients, each with at least 5 time points that includes key milestones: pre-surgery, short-term (2 weeks–1 month) and long-term post-surgery (3–12 months).

## Results

### Multiplex Amplification of Pancreatic Specific cfmRNA From Plasma

Circulating cell free mRNA (cfmRNA) has been shown to contain transcripts that are derived from multiple organs and can be used to capture tissue-specific transcriptional change. Many of these cell free RNA transcriptome sequencing studies have also shown that tissue-specific circulating RNA can present at very low levels in the plasma (Koh et al., 2014; Toden et al., 2020). To address the potentially low levels of pancreatic specific RNA in plasma, we chose a targeted approach and integrated three highly sensitive multiplex PCR strategies [RNaseH2-dependent PCR (Crissman et al., 2020), Emulsion PCR (Williams et al., 2006) and CoT PCR (Gijavanekar et al., 2012)] from other studies into a single workflow.

**FIGURE 1:**
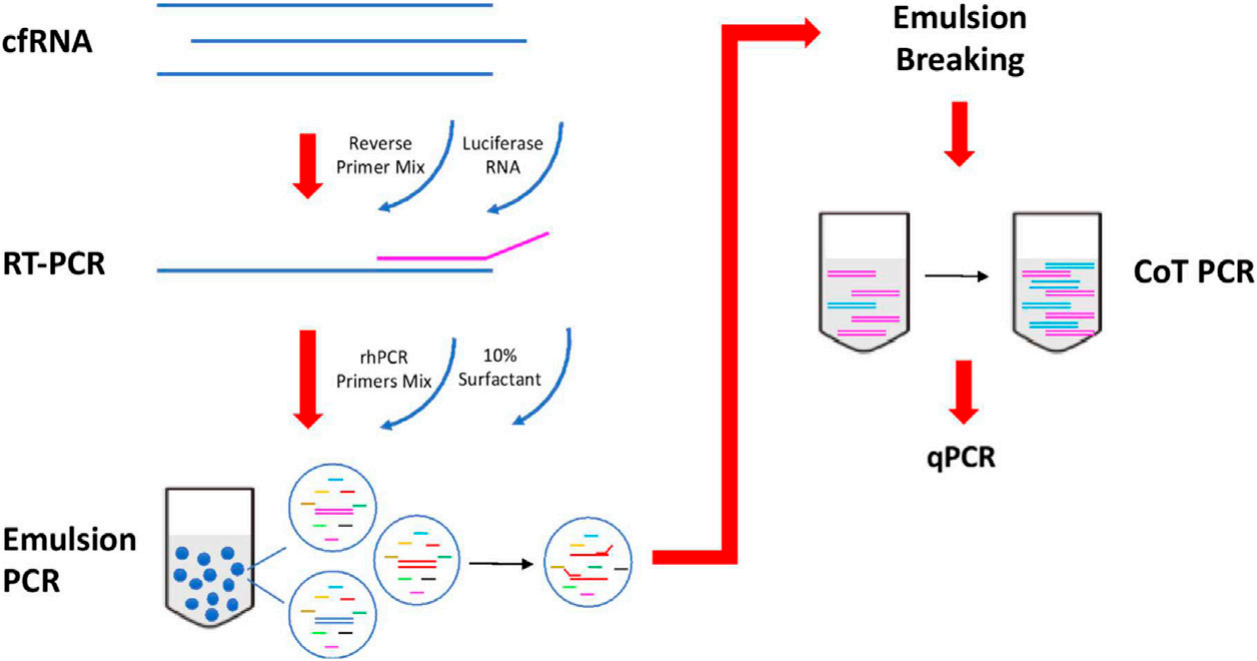
Workflow of reverse transcription and amplification of cfmRNA. cfmRNA, spiked-in with luciferase RNA control, was reverse transcript with Superscript™ III Reverse Transcriptase (Invitrogen, Cat no. 18080044). The product was amplified with rhPCR primers and Platinum™ Taq DNA Polymerase (Invitrogen, Cat no. 10966) using emulsion and CoT PCR. The residual primers were removed with Exonuclease I (New England Biolabs, Cat no. M0293). Amplified products were used for qPCR quantification and sequencing.

An overview of the circulatory RNA amplification workflow is presented in Figure 1. The first step is deploying multiplexed pre-amplification using RNaseH2-dependent PCR (rhPCR) after the initial reverse transcription of cfRNA. Exon-spanning rhPCR primers (rhPrimers) were designed for each of our 14 pancreatic gene targets. Each rhPrimers contains a single RNA base and are used in conjunction with thermostable RNase H2 enzyme to perform rhPCR. When rhPrimer anneal to the intended pancreatic specific target cDNA, a substrate for RNaseH2 is formed at the site where the single RNA residue resides in the primer. When there is perfect complementarity, RNaseH2 cleaves the RNA site, removing the blocking group from the primer, facilitating DNA synthesis. Imperfect complementarity does not result in this unblocking reaction. This result in improved specificity of the priming event and allows for our targeted multiplex cDNA amplification.

To further reduce the formation of any unwanted chimeric products, the rhPCR step is performed using emulsion based PCR technique where aqueous rhPCR mix is compartmentalized into an oil-water emulsion before thermocycling. The resulting amplicons are subsequently recovered by freezing the mix at −80C which breaks the emulsion. Recovered amplicons are then put through CoT PCR enrichment that preferentially amplifies rare amplicons over abundant ones by taking advantage of the CoT effect (Mathieu-Daudé et al., 1996).

Additional housekeeping genes (ACTB, GAPDH, RPS18) and Luciferase (LUC) gene are included as quality control targets in the assay to ensure that results are comparable across plasma samples after amplification. Quantification of housekeep genes is used for normalization of cfmRNA extraction efficiency. Moreover, each of the extracted cfmRNA samples are also spiked with a 10^5^ molecules of commercially obtained Luciferase Control RNA which is used to normalize technical variability arising from the amplification process.

### Screening for Temporal Changes in Circulating Pancreatic cfmRNA

**FIGURE 2:**
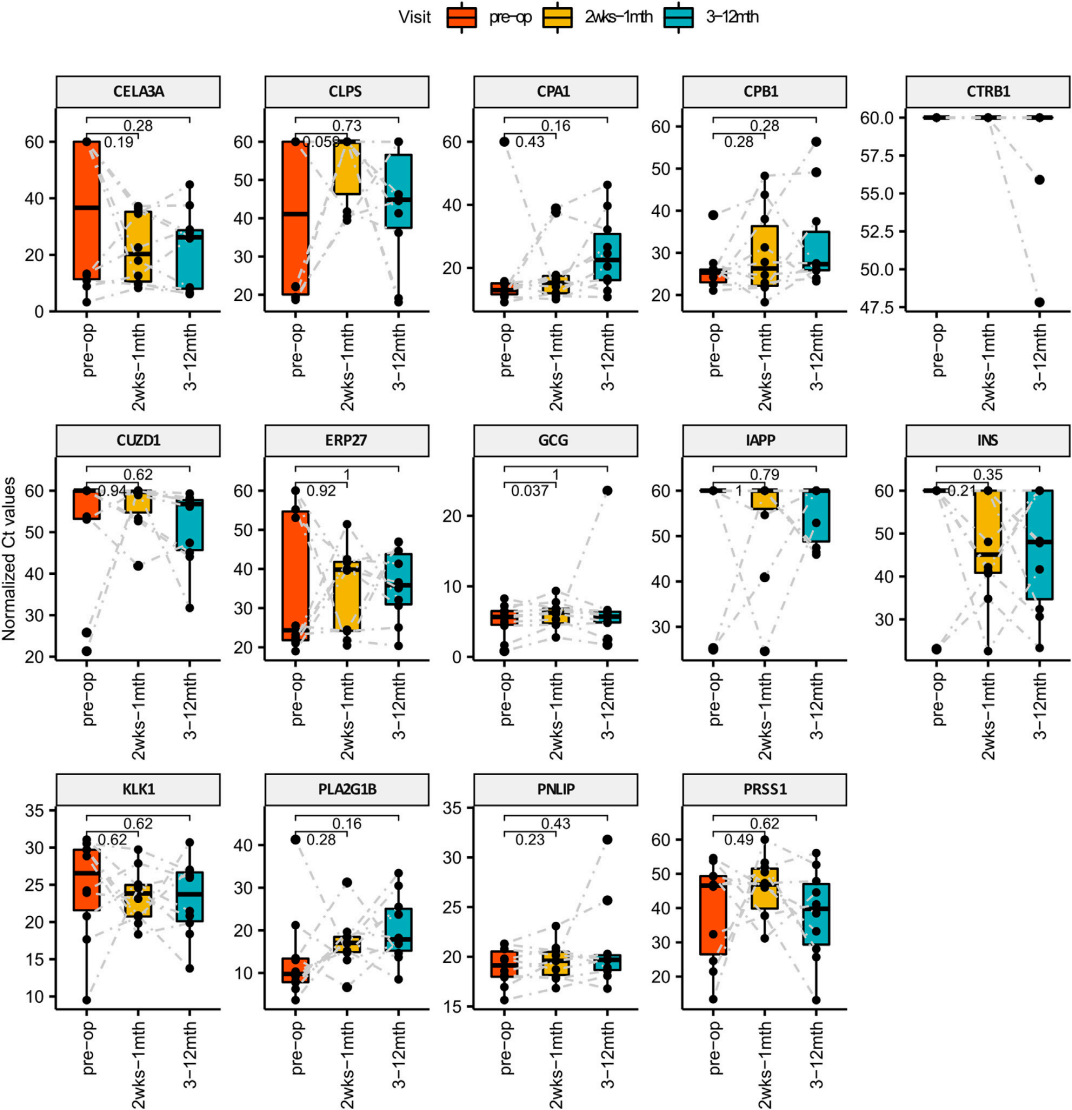
Bar Charts comparing the levels of pancreatic specific transcripts across different visit post-surgery. *X*-axis represents the different time points and the *Y*-axis is the normalized Ct value of the cfmRNA. Wilcox paired test is used for statistically comparing the short and long term levels of cfmRNA against the pre-surgery measurements. GCG cfmRNA Ct measurements was identified to be statistically significant higher in the short term post-surgery which translates to a lower quantity of GCG cfmRNA in circulation immediately after bariatric surgery.

Plasma was collected from each patient across at least 5 different visits that spans 3 main phases: Pre-surgery, Short-term post-surgery (2 weeks–1 month) and Long-term post-surgery (3–12 months). Circulating RNA extracted from 1 ml of plasma was quantified for pancreatic specific transcripts using the methods described. We found that one of the pancreatic transcripts CTRB1 (Chymotrypsinogen B1) did not amplified from plasma and hence not detected using qPCR. Of the remaining 13 transcripts that were amplified, we performed the Wilcoxon paired test comparing the qPCR Ct values of each pancreatic specific transcript pairwise across different visits i.e., 1) pre-surgery vs short-term post-surgery, 2) pre-surgery vs. long-term post-surgery as shown in Figure 2. Although temporal variations are observed amongst the remaining 13 amplifiable transcripts within individual patients, changes in GCG level is the most statistically consistent (*p*-value: 0.037) when comparing pre-surgery vs short-term post-surgery levels. Higher Ct levels are observed for circulating GCG transcripts during the short-term post-surgery period which translates to a decrease in amount of circulating GCG transcripts during the period immediately post-surgery.

### GCG cfmRNA Levels Correlates With Common Clinical Biomarkers Used to Monitor Bariatric Surgery

**FIGURE 3:**
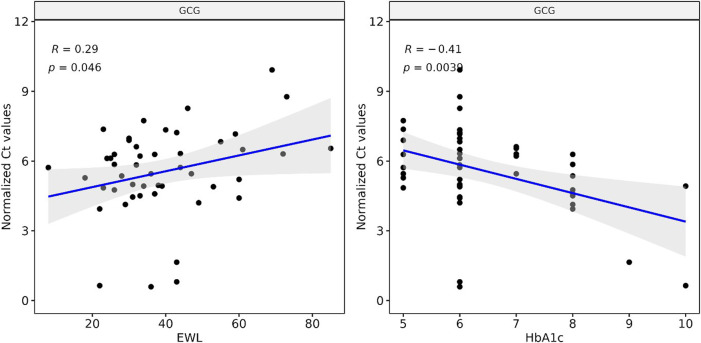
Levels of GCG cfmRNA correlates with clinical biomarkers HbA1c and %EWL (Percent excess weight loss) Ct measurements of GCG cfmRNA across all patients and visits are correlated with %EWL and HbA1c levels. Linear regression captures a significant positive correlation with %EWL and an inverse correlation with HbA1c.

We correlated our Ct measurements of GCG cfmRNA against clinical biomarkers collected routinely to track the outcome of bariatric surgery. GCG cfmRNA Ct measurements correlates positively (R: 0.29, *p*-value: 0.046) with percentage excess weight loss (%EWL) and exhibit an inverse correlation (R: −0.41, *p*-value: 0.0039) with glycated haemoglobin (HbA1c) levels as shown in Figure 3. %EWL is a common metric for quantifying weight loss due to bariatric surgery whereas HbA1c is typically used to quantify metabolic outcomes such as the extent of diabetes. Here we noticed that levels of GCG cfmRNA captures both weight loss and metabolic outcome metrics and could potentially serve as an additional metric for monitoring surgical outcomes.

GCG gene expression is pivotal for metabolic regulation. The gene encodes for multiple peptides including glucagon, glucagon-like peptide-1, glucagon-like peptide-2, oxyntomodulin, and glicentin, and is shown to be expressed primarily in pancreatic α-cells, and L-cells of the ileum and large intestine. It is well established that the control of GCG transcription is under nutrient and hormonal control, and can exhibit cell type specific regulation. Studies using animal models have previously implicated expression differences in both α- and L-cells types following bariatric surgery (Rhee et al., 2015; Ribeiro-Parenti et al., 2021). In general, circulating cell free RNA levels are dependent on both expression levels and the apoptotic rate of the cells releasing nucleic acid into the circulatory system. The captured correlation and temporal trends in GCG cfmRNA levels captured in our assay could be suggestive of either transcriptomic changes, reduced rate of pancreatic cell death or both.

## Discussions

Numerous high throughout studies have demonstrated the potential of cfmRNA as biomarkers especially in the field of cancer diagnostics in the form of liquid biopsies. Ibarra et al. (2020), Toden et al. (2020), Chalasani et al. (2021), Larson et al. (2021) However, few have explored the utility of tissue-specific cfmRNA as biomarkers for metabolic outcomes. In our study, we demonstrated that a multiplex RT-qPCR approach can be used for the amplification and quantification of tissue-specific cfmRNA, especially those of pancreatic specificity. Tissue-specific cfmRNAs comprised of genes that are expressed at higher levels in the specified subset of tissues relative to the baseline expression across all tissues. (Koh et al., 2014) These tissue-specific genes often play critical roles in maintaining biological functions unique to those tissues. Recent studies have also established that changes in tissue-specific cfmRNA levels correlates to progression in diseases such as Alzheimer’s disease where a significant portion of dysregulated cfmRNA are found to be brain-specific. (Toden et al., 2020) Within the metabolic context of bariatric surgery, we postulate that post-surgical improvements in glucose homeostasis after surgery can be captured by characterizing pancreatic-specific cfmRNA. By focusing on the subset of pancreatic-specific genes identified by the Human Protein Atlas (Uhlen et al., 2010), the multiplex RT-qPCR approach was able to simultaneously amplified and quantitated 13 out of 14 intended pancreatic specific cfmRNA from individual plasma sample. As the plasma-based cfmRNA assay is non-invasive in nature, we were able to make multiple measurements: pre and post-surgery. Temporal analysis of Ct values obtained from screening pancreatic-specific cfmRNA lead to the identification of a small but consistent decrease in levels of circulating GCG cfmRNA during the short-term post-surgery. Expanding on the GCG cfmRNA target, we observed significant correlations of GCG cfmRNA to other clinical measurements (%EWL and HbA1c) obtain at across sampled time points. Although the observed trends suggests that metabolic changes in pancreas can be captured *via* the plasma, more studies especially those exploring other surgical interventions will be needed to confirm the mechanism behind the source of GCG cfmRNA in plasma which most likely originate from α or L cells (Panaro et al., 2020). We also recognise that the range of change obtained using Ct measurements for cfmRNA levels can be small in our study. Future work can build upon these initial changes identified and employ higher sensitive methods such as digital PCR for direct molecular counting of GCG cfmRNA. Nevertheless, the demonstrated ability to survey multiple gene targets of pancreatic specificity indicate that such a multiplex RT-qPCR approach can be used as an affordable proxy for non-invasive molecular evaluation of tissue specific molecular alterations in patients with metabolic diseases.

We also note that our current proof of concept study has several limitations especially in terms of the cohort size, the frequency of temporal sampling as well as the number of circulating RNA targeted. Despite these qualifications, our data supports the notion that sufficient pancreatic specific cfmRNA can be amplified from plasma and potentially be used as a tool to identify transcriptional alterations of the pancreas that might otherwise be difficult to access and monitor. Furthermore, our protocol highlights an alternative to whole cfmRNA transcriptome sequencing approaches, by focusing only on pancreatic specific cfmRNA, the approach allows for much affordable measurements *via* qPCR and subsequently reduce the cost of deployment for scalable applications such as patient stratification.

## Materials and Methods

### Tissue Specific Gene Selection Using Human Protein Atlas

Genes that are specific to the Pancreas were selected based on the Human Protein Atlas (https://www.proteinatlas.org/). Primers were designed and validated to amplify cross-exons region by using UCSC Genome Browser in-silico PCR tool. (https://genome.ucsc.edu/).

### Plasma Sample Collection

Blood samples were acquired though National University Hospital following DSRB approval. Whole blood was subjected to 2 stage centrifugation (1600G and 16000G) within a day after collection and the plasma collected were stored in 1 ml aliquots at −80C before RNA extraction. Clinical measurements such as percentage excess weight loss (%EWL = (baseline weight–follow-up visit weight)/(baseline weight—IBW) * 100.) and glycated haemoglobin (HbA1c) levels are also collected alongside each plasma sample collection at every visit.

### Primer Design

Rnase H-dependent PCR (rhPCR) primers was designed according to Integrated DNA Technologies Gen1 design. The primer consists of 5 different parts starting at the 5’ end with the final functional primer (comprising of more than 10 DNA bases that matches the template), the cleavage site (single RNA residue), four matching DNA bases, one mismatch DNA base, and lastly the blocking group (C3 spacer) at the 3’ end. All primers sequences are provided in the Supplementary Table S1.

### Circulating RNA Extraction

cfmRNA was extracted from 1 ml of plasma using Plasma/Serum Circulating and Exosomal RNA Purification Kit (Norgen, Cat no. 42800). The residual DNA in the cfmRNA was digested using RNase-Free DNase I Kit (Norgen, Cat no. 25720). Extracted cfmRNA was purified using RNA Clean and Concentrator™-5 (Zymo, Cat no. ZYR. R1016), yielding 24 μL of cfRNA per sample.

### Spiked-In Quality Controls

10^5^ copies of Luciferase Control RNA (Promega, Cat no. L4561) was spiked in with 10 μL of the extracted cfRNA at the reverse transcription step. It was used as a control to normalise for any unintended variation in the experiment.

### Reverse Transcription and Emulsion Based Targeted Pre-amplification

10 μL of extracted cfRNA was annealed with a final concentration of 0.4 μM of reverse primers mix in the presence of 10^5^ copies of luciferase control RNA (Promega, Cat no. L4561) and a final concentration 2 mM of dNTPs at 65°C for 5 min. Reverse transcription of cfRNA was performed using Superscript™ III Reverse Transcriptase (Invitrogen, Cat no. 18080044) at 25°C for 5 min, 50°C for 50 min, and enzyme inactivation at 95°C for 3 min.

cDNA from reverse transcription was added to the PCR mixture of Platinum™ Taq DNA Polymerase (Invitrogen, Cat no. 10966) with a final concentration of 0.5 μM of rh PCR primers mix and 26 mU of RNase H2 enzyme. Emulsion was generated by adding 3 parts of 10% 008-FluoroSurfactant (RAN Biotechnologies) in 3M Fluorinert ™ Engineered Fluid (3M, Cat no. FC-40) to 1 part of PCR reaction mixture. The mixture was vortexed until it became cloudy and uniform. Thermocycling started with enzyme activation at 94°C for 2 min, followed by 20 cycles of denaturation (94°C, 15 s), annealing (55°C, 30 s), and extension (68°C, 1 min). Reaction recovered from emulsion PCR was top up with the same amount of polymerase and RNase H2 used in emulsion PCR. Thermocycling started with enzyme activation at 94°C for 2 min, followed by 20 cycles of denaturation (94°C, 15 s), hybridization (78°C, 10 min), annealing (55°C, 30 s), and extension (68°C, 1 min).

### Quantification of Pre-amplified Gene Targets Using qPCR

To monitor the expression of the targeted cfRNA across the different time point, qPCR was performed for 60 cycles with Maxima SYBR Green/ROX qPCR Master Mix (ThermoFisher Scientific, Cat no. K0221). 1 ul of the amplified target, 0.3 uM of the forward and reverse primers are added to 12.5 ul of the master mix. Water is then added to form a total 25 ul reaction volume. The mix is then run on the QuantStudio 5 qPCR system using the thermal profile: 95°C for 10 min for the initial denaturation, followed by 60 cycles of 95°C denaturation (15 s) and 65°C annealing and extension (60 s). Melting curve analysis is also performed by ramping the temperature up to 95°C after the qPCR to verify the specificity and identity of each of the PCR product.

### Data Analysis

Melting temperatures of the circulating pancreatic transcripts are compared to the reference melting temperature of the amplicons obtained from using commercially obtained RNA (Takara Bio) extracted from pancreatic tissue. Mismatch in amplicon melting temperature indicates a wrong amplification product and the corresponding Ct value will be set to 60 which is the maximum number of cycles.

Ct values of the transcripts then undergo two normalization steps. The first normalization step is to normalize to the Ct value of the spiked in luciferase which will account for technical variation during the PCR steps across different samples. The second normalization step uses the geometric mean of the housekeep genes and is meant to account for variation in the amount of extracted RNA. Paired wilcox test is then performed on the normalized Ct values comparing the transcript levels pre-surgery and post-surgery (short and long term). All melt curves and amplification plots are included as Supplementary Figures S1,S2.

## Data Availability

The original contributions presented in the study are included in the article/Supplementary Material, further inquiries can be directed to the corresponding authors.

## Supplementary Material

Supplementary Figure S1 Melt curves of all cfmRNA targets. Each patient is sampled at multiple visits and each plasma sample is tested against a panel of housekeep (Left Panel) and pancreatic targets (RightPanel). Each column is a different qPCR target and each row of the plot corresponds to a single patient sample and the colour correspond to different time points from that patient. The x-axis correspond to temperature and the y-axis corresponds to the derivative reporter that captures the rate of change in fluorescence during the temperature ramp. During amplification when the amplicon is successfully amplified, the peak of the derivative reporter is expected to coincide with the designed melting temperature (vertical line) and expected to be similar across all samples and time-points. These properties are used as a quality control measure step in the study.

Supplementary Figure S2 Raw Amplification Curves of qPCR targets. Each patient is sampled at multiple visits and each plasma sample is tested against a panel of housekeep (Left Panel) and pancreatic targets (Right Panel). Here each column is a different qPCR target and each row of the plot corresponds to a single patient sample and the colour correspond to different time points from a particular patient. The x-axis corresponds to cycle number and the y-axis correspond to delta Rn obtained by dividing the fluorescence emission intensity of the reporter dye by the fluorescence emission intensity of the passive reference dye. Ct values obtained from these raw amplification curves are subsequently normalized using the housekeep genes before comparison across time points.

Supplementary Table S1 Table of Primers used in targeted amplification and quantification of pancreatic assay. The primers used are categorize into 3 main groups with different targets: Pancreas, Housekeeping and Quality Control. Within each main group, primers are listed according to the individual targeted gene. The reverse primers listed for each target are pooled together for use in the initial reverse transcription step. Following this, rhPCR preamplification is performed using the pool of pairs of reverse and forward rhprimers. The last column contain the reverse and forward primer design for individual qPCR assay to quantify the amount of transcripts in the amplified cDNA products.
