# Supplementary material for: Non-Invasive Characterization of the Pancreas During Bariatric Surgery via Circulating Pancreatic Specific Cell-free Messenger RNA
Source: Front Genet. 2021 Oct 11;12:742496. doi: 10.3389/fgene.2021.742496 (PMC8542674; doi:10.3389/fgene.2021.742496)
Supplement: Supplementary file 1 [file table1.docx]

| **Category** | **Targets** | **Reverse Primers** | **rh Primers** | **qPCR Primers** |
| --- | --- | --- | --- | --- |
| Pancreas | CTRB1 Forward |  | CAAGTACAACGCCAACAAGACrCCCTGG/3SpC3/ | CAAGTACAACGCCAACAAGAC |
|  | CTRB1 Reverse | GTCCAGGCTCCATCCTTTT | GTCCAGGCTCCATCCTTTTrGGCAGT/3SpC3/ | GTCCAGGCTCCATCCTTTT |
|  | IAPP Forward |  | AATGGGCATCCTGAAGCTGrCAAGTT/3SpC3/ | AATGGGCATCCTGAAGCTG |
|  | IAPP Reverse | CATGTGGCAGTGTTGCA | CATGTGGCAGTGTTGCATTTrCCGCTA/3SpC3/ | CATGTGGCAGTGTTGCA |
|  | CELA3A Forward |  | ACCATGGTGTGTGCTGGAGrGGTACC/3SpC3/ | ACCATGGTGTGTGCTGGAG |
|  | CELA3A Reverse | AACAAAGCTGGTCACACC | AACAAAGCTGGTCACACCGTrGGACCA/3SpC3/ | AACAAAGCTGGTCACACC |
|  | PNLIP Forward |  | GGGAAAACAAATGATGTGGGrCCAGAG/3SpC3/ | GGGAAAACAAATGATGTGGG |
|  | PNLIP Reverse | TTTCCAGACAGTGTGACAGATACC | TTTCCAGACAGTGTGACAGATACCrTTATAG/3SpC3/ | TTTCCAGACAGTGTGACAGATACC |
|  | CPB1 Forward |  | TCAAGGCATATCTGACAATCCArCTCGTT/3SpC3/ | TCAAGGCATATCTGACAATCC |
|  | CPB1 Reverse | CAGTAGCTTTAGCCAGGGCA | CAGTAGCTTTAGCCAGGGCArTTCAAG/3SpC3/ | CAGTAGCTTTAGCCAGGGCA |
|  | CLPS Forward |  | TCCTGATCCTCCTGCTTGTCrGCCCTT/3SpC3/ | TCCTGATCCTCCTGCTTGTC |
|  | CLPS Reverse | TCATGCAGAGCTCACCGTT | TCATGCAGAGCTCACCGTTrCTCCTC/3SpC3/ | TCATGCAGAGCTCACCGTT |
|  | PRSS1 Forward |  | TGTGGTGGCTCCCTCATCAArCGAACT/3SpC3/ | ATCAACGAACAGTGGGTGGT |
|  | PRSS1 Reverse | CACGCGGGCGTTGATTACT | CACGCGGGCGTTGATTACTrGCACGA/3SpC3/ | CTCATTCCCCTCCAGGACTT |
|  | PLA2G1B Forward |  | GAAACTCCTTGTGCTAGCTGTGrCTGCTG/3SpC3/ | TTGTGCTAGCTGTGCTGCTC |
|  | PLA2G1B Reverse | GTAGCAGCCGTAGTTGTTGT | GTAGCAGCCGTAGTTGTTGTrATTCCT/3SpC3/ | GCACTTGATCATTTTGCGG |
|  | CUZD1 Forward |  | GGCTCATGCCATTGACCCTCrUTAATG/3SpC3/ | ATTCTCTCCTGTTTGGCGG |
|  | CUZD1 Reverse | ACTGGGATTGAGTTGCAGGA | ACTGGGATTGAGTTGCAGGArUCATGC/3SpC3/ | TCATGGCTTTGTGGGTCTCT |
|  | GCG Forward |  | CACAGGGCACATTCACCAGTrGACTAG/3SpC3/ | CACAGGGCACATTCACCAGT |
|  | GCG Reverse | AATTCATCGTGACGTTTGGC | AATTCATCGTGACGTTTGGCrAATGTA/3SpC3/ | AATTCATCGTGACGTTTGGC |
|  | CPA1 Forward |  | GCAAGATCCAGATTGGCAACrACCTAG/3SpC3/ | GCAAGATCCAGATTGGCAAC |
|  | CPA1 Reverse | CAGATGGCTGGACGCTTACT | CAGATGGCTGGACGCTTACTrGCCCCG/3SpC3/ | CAGATGGCTGGACGCTTACT |
|  | KLK1 Forward |  | TTTCCAGTGTGGGGGCATCrCTGGTC/3SpC3/ | CTCACAGCTGCTCATTGCAT |
|  | KLK1 Reverse | CATGTTGAAGCCAGGGTGTG | CATGTTGAAGCCAGGGTGTGrGGAAGG/3SpC3/ | AACTGGGCTGTGTTTTCGTC |
|  | ERP27 Forward |  | TGCCACCAAATTGAGCCGTrUTCATG/3SpC3/ | CCTCCACATGGTGACAGAGTACA |
|  | ERP27 Reverse | CTTGGCTGCCTTCTGGTATCT | CTTGGCTGCCTTCTGGTATCTrGTGCAA/3SpC3/ | CTCTTCATACTCTGGGGAGGC |
|  | INS Forward |  | CATCTGCTCCCTCTACCArGCTGGG/3SpC3/ | CATCTGCTCCCTCTACCA |
|  | INS Reverse | CTTTATTCCATCTCTCTCGGT | CTTTATTCCATCTCTCTCGGTrGCAGGT/3SpC3/ | CTTTATTCCATCTCTCTCGGT |
| Housekeeping | GAPDH Forward |  | ATGTTCGTCATGGGTGTGrAACCAG/3SpC3/ | AGAAGTATGACAACAGCCTCA |
|  | GAPDH Reverse | CATGAGTCCTTCCACGATA | CATGAGTCCTTCCACGATArCCAAAC/3SpC3/ | CCAAAGTTGTCATGGATGA |
|  | ACTB Forward |  | AGAAGAGCTACGAGCTGCCTGArCGGCCC/3SpC3/ | CATCACCATTGGCAATGA |
|  | ACTB Reverse | TGGAGTTGAAGGTAGTTTCG | TGGAGTTGAAGGTAGTTTCGrUGGATT/3SpC3/ | AAGGTAGTTTCGTGGATGC |
|  | RPS18 Forward |  | TATGCTCATGTGGTGTTGAGrGAAAGG/3SpC3/ | AGACATTGACCTCACCAAGA |
|  | RPS18 Reverse | AAGAACCAGTCTGGGATCTT | AAGAACCAGTCTGGGATCTTrGTACTC/3SpC3/ | TTGTACTGGCGTGGATTC |
| Control | LUC Forward |  | CTCTGATTAACGCCCAGCrGTTTTG/3SpC3/ | CTCTGATTAACGCCCAGC |
|  | LUC Reverse | GCGGTCGGTAAAGTTGTT | GCGGTCGGTAAAGTTGTTrCCATTA/3SpC3/ | GCGGTCGGTAAAGTTGTT |

Supplementary Table 1. **Table of Primers used in targeted amplification and quantification of pancreatic assay.** The primers used are categorize into 3 main groups with different targets: Pancreas, Housekeeping and Quality Control. Within each main group, primers are listed according to the individual targeted gene. The reverse primers listed for each target are pooled together for use in the initial reverse transcription step. Following this, rhPCR preamplification is performed using the pool of pairs of reverse and forward rhprimers. The last column contain the reverse and forward primer design for individual qPCR assay to quantify the amount of transcripts in the amplified cDNA products.
