# Supplementary material for: Non-Invasive Characterization of the Pancreas During Bariatric Surgery via Circulating Pancreatic Specific Cell-free Messenger RNA
Source: Front Genet. 2021 Oct 11;12:742496. doi: 10.3389/fgene.2021.742496 (PMC8542674; doi:10.3389/fgene.2021.742496)
Supplement: Supplementary file 2 [file presentation1.pptx]

## Slide 1
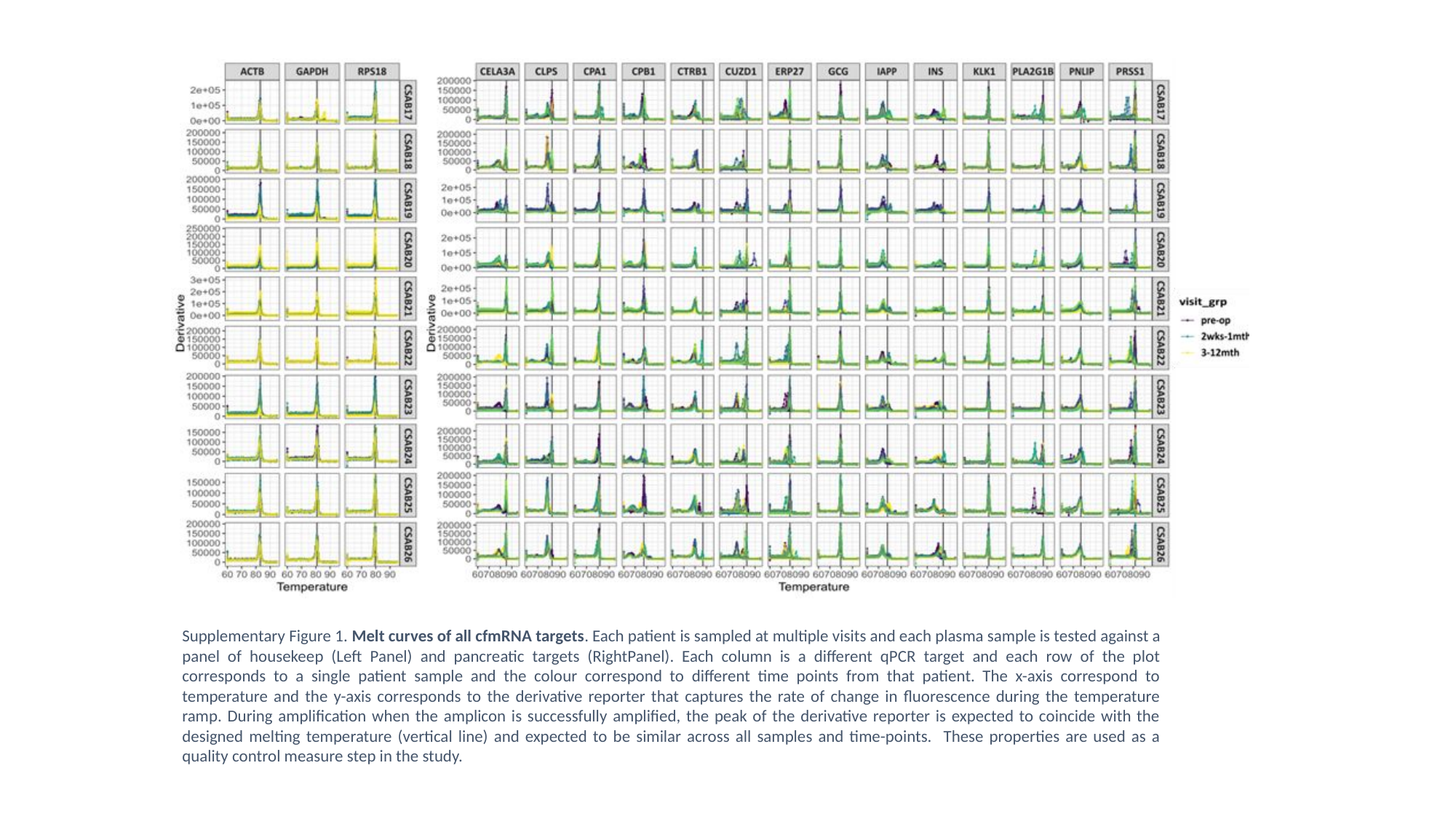

Supplementary Figure 1. Melt curves of all cfmRNA targets. Each patient is sampled at multiple visits and each plasma sample is tested against a panel of housekeep (Left Panel) and pancreatic targets (RightPanel). Each column is a different qPCR target and each row of the plot corresponds to a single patient sample and the colour correspond to different time points from that patient. The x-axis correspond to temperature and the y-axis corresponds to the derivative reporter that captures the rate of change in fluorescence during the temperature ramp. During amplification when the amplicon is successfully amplified, the peak of the derivative reporter is expected to coincide with the designed melting temperature (vertical line) and expected to be similar across all samples and time-points. These properties are used as a quality control measure step in the study.

## Slide 2
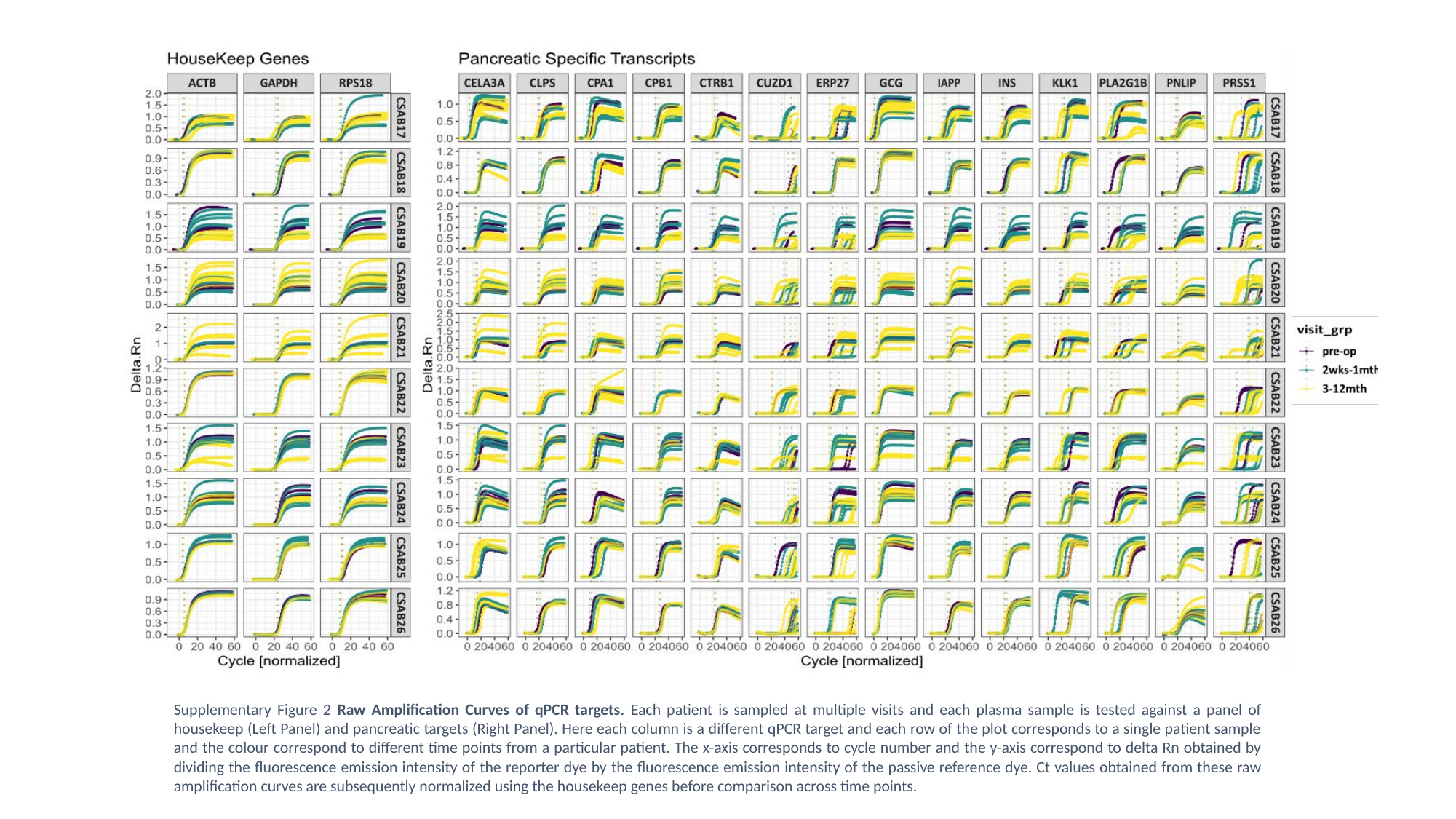

Supplementary Figure 2 Raw Amplification Curves of qPCR targets. Each patient is sampled at multiple visits and each plasma sample is tested against a panel of housekeep (Left Panel) and pancreatic targets (Right Panel). Here each column is a different qPCR target and each row of the plot corresponds to a single patient sample and the colour correspond to different time points from a particular patient. The x-axis corresponds to cycle number and the y-axis correspond to delta Rn obtained by dividing the fluorescence emission intensity of the reporter dye by the fluorescence emission intensity of the passive reference dye. Ct values obtained from these raw amplification curves are subsequently normalized using the housekeep genes before comparison across time points.
